# Robust Archaeal and Bacterial Communities Inhabit Shallow Subsurface Sediments of the Bonneville Salt Flats

**DOI:** 10.1128/mSphere.00378-19

**Published:** 2019-08-28

**Authors:** Julia M. McGonigle, Jeremiah A. Bernau, Brenda B. Bowen, William J. Brazelton

**Affiliations:** a School of Biological Sciences, University of Utah, Salt Lake City, Utah, USA; b Department of Geology and Geophysics, University of Utah, Salt Lake City, Utah, USA; National Institute of Advanced Industrial Science and Technology, Japan

**Keywords:** astrobiology, extremophiles, halophiles, hypersaline environments

## Abstract

We report the first census of natural microbial communities of the Bonneville Salt Flats (BSF), a perennial salt pan at the Utah-Nevada border. Environmental DNA sequencing of archaeal and bacterial 16S rRNA genes was conducted on samples from multiple evaporite sediment layers collected from the upper 30 cm of the surface salt crust. Our results show that at the time of sampling (September 2016), BSF hosted a robust microbial community dominated by diverse halobacteria and *Salinibacter* species. Sequences identical to *Geitlerinema* sp. strain PCC 9228, an anoxygenic cyanobacterium that uses sulfide as the electron donor for photosynthesis, are also abundant in many samples. We identified taxonomic groups enriched in each layer of the salt crust sediment and revealed that the upper gypsum sediment layer found immediately under the uppermost surface halite contains a robust microbial community. In these sediments, we found an increased presence of *Thermoplasmatales*, *Hadesarchaeota*, *Nanoarchaeaeota*, *Acetothermia*, *Desulfovermiculus*, *Halanaerobiales*, *Bacteroidetes*, and *Rhodovibrio*. This study provides insight into the diversity, spatial heterogeneity, and geologic context of a surprisingly complex microbial ecosystem within this macroscopically sterile landscape.

**IMPORTANCE** Pleistocene Lake Bonneville, which covered a third of Utah, desiccated approximately 13,000 years ago, leaving behind the Bonneville Salt Flats (BSF) in the Utah West Desert. The potash salts that saturate BSF basin are extracted and sold as an additive for agricultural fertilizers. The salt crust is a well-known recreational and economic commodity, but the biological interactions with the salt crust have not been studied. This study is the first geospatial analysis of microbially diverse populations at this site using cultivation-independent environmental DNA sequencing methods. Identification of the microbes present within this unique, dynamic, and valued sedimentary evaporite environment is an important step toward understanding the potential consequences of perturbations to the microbial ecology on the surrounding landscape and ecosystem.

## Abstract (precis)

Pleistocene Lake Bonneville, which covered a third of Utah, desiccated approximately 13,000 years ago, leaving behind the Bonneville Salt Flats (BSF) in the Utah West Desert. The potash salts that saturate BSF basin are extracted and sold as an additive for agricultural fertilizers. The salt crust is a well-known recreational and economic commodity, but the biological interactions with the salt crust have not been studied. This study is the first geospatial analysis of microbially diverse populations at this site using cultivation-independent environmental DNA sequencing methods. Identification of the microbes present within this unique, dynamic, and valued sedimentary evaporite environment is an important step toward understanding the potential consequences of perturbations to the microbial ecology on the surrounding landscape and ecosystem.

## INTRODUCTION

The Great Salt Lake and Bonneville Salt Flats (BSF) are both remnants of Pleistocene Lake Bonneville which partially drained at the end of the last glacial maximum and desiccated ∼13,000 years ago (1). BSF is famous for its use as a speedway through the last century, which has hosted many land-speed records and a community that greatly values this saline landscape (2). Over this same time period, the salts that saturate BSF basin have been extracted as an economic commodity, particularly as potash (e.g., KCl) that is sold as an additive for agricultural fertilizers. The hydrology and sedimentology of BSF have been studied periodically throughout the 20th century in relation to potash mining and concerns about the impacts of land use on the environment (3–9). Human land use has altered many aspects of the hydrology and morphology of the environment that facilitated deposition of the ∼2-m-thick evaporite lens-shaped crust that caps BSF. The amount and extent of salt present at the site have been observed to fluctuate and decrease through the past century (10, 11).

Examination of modern and ancient salt pan deposits demonstrates that these environments undergo repeated cycles of desiccation (dry saline pan), flooding (brackish lake), and evaporative concentration (drying to dry saline pan) (12, 13). The desiccation stage is repeatedly interrupted by flooding events related to individual storms, wet seasons, and spring thaws driving runoff from surrounding mountains. The environmental parameters that influence the hydrology and timing of flooding, evaporation, and desiccation cycles are dynamic (14). For this study, multiple individual strata from several sites spanning the salt crust were sampled at BSF during an extensive sampling campaign to measure the overall stratigraphy and volume of the salt crust (11). The samples were collected in early September 2016 near the end of a 3-month desiccation period.

Microbial communities provide critical ecosystem services by cycling nutrients and providing a biological foundation upon which other organisms can establish themselves. In environments considered “extreme” by human standards such as BSF, microbes are often the only life forms that can survive. The study of microbial life in these ecosystems can elucidate how extremophilic organisms have evolved unique attributes, strategies, and metabolic capabilities that enable them to thrive where most other organisms cannot (15, 16). Extreme hypersaline ecosystems select for organisms capable of not only tolerating, but actually requiring high levels of salt for growth (17). Microbes in these ecosystems employ either a “salt-in” or “organic-solutes-in” strategy to overcome the problems associated with osmoregulation in a high-salt environment (18). In addition, halophiles found in these environments produce enzymes with potential biotechnological, bioremediation, and medical applications (19).

The role of microbes in BSF salt crust processes are generally unknown. Identification of the microbes present within this unique, dynamic, and valued evaporite environment is an important step toward understanding the potential consequences of perturbations to the microbial ecology on the surrounding landscape and ecosystem. This study is the first geospatial analysis of microbially diverse populations at BSF using cultivation-independent environmental DNA sequencing methods. This study provides insight into the diversity, spatial heterogeneity, and geologic context of a surprisingly complex microbial ecosystem within this macroscopically sterile landscape.

## RESULTS

### Elemental and sedimentological analysis.

Thin sections and reconstructed mineralogy from elemental data were used to group each sample into one of four classifications: group 1 (surface halite), group 2 (upper gypsum), group 3 (lower halite), and group 4 (halite mixed with gypsum).

**FIG 1:**
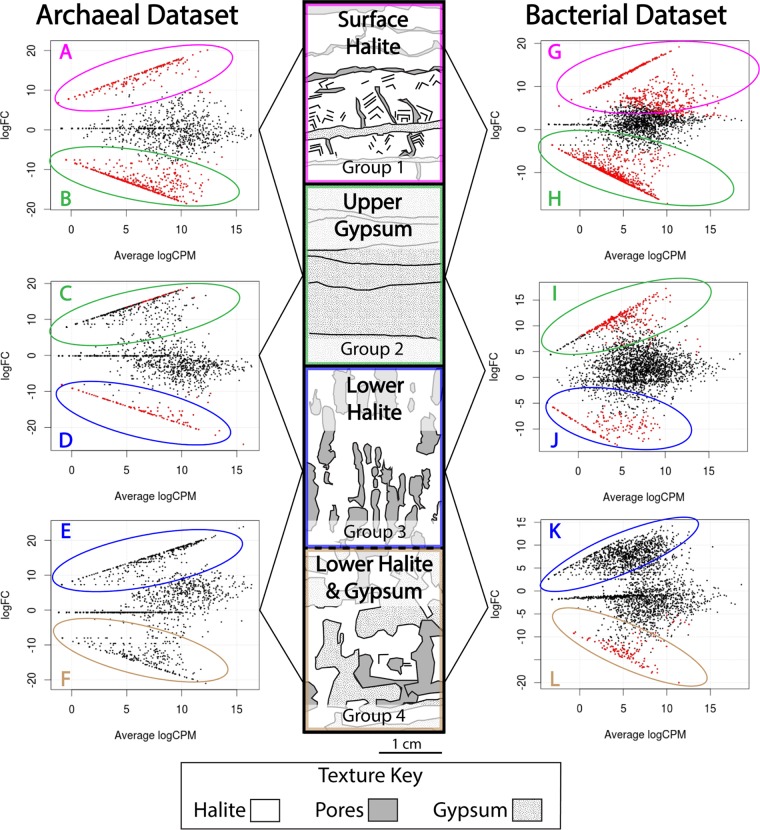
Sedimentology and differential abundance results (edgeR) for archaeal and bacterial data sets. Red dots indicate an ASV with a significantly higher abundance (FDR < 0.05) in the indicated category. Representative stratigraphic columns are presented here. Dashed lines represent stratigraphic shifts that only occurred at some sites. General stratigraphy consists of groups 1 to 5. Group 1, surface halite, contains efflorescent halite and halite crystals with abundant fluid inclusions. Group 2, upper gypsum, consists of gypsum grains with various proportions of halite; this group has the highest proportion of clays. Group 3, lower halite, has the highest proportion of pore space and is mineralogically similar to group 1. Group 4, lower halite and gypsum, forms when pores in lower halite fill with gypsum. Axis abbreviations: logFC, log_2_-fold change (differential abundance); logCPM, log_2_ counts per million across all samples in comparison.

Elemental analysis and thin section microscopy reveal layers of distinct sedimentological morphology (Fig. 1; see also Fig. S2 in the supplemental material). Primary minerals were identified as halite (NaCl), gypsum (CaSO_4_ ⋅ 2H_2_O), and clay and detrital minerals (Fe, Mg, and Al). The majority of samples were above reporting limits for sodium. All minerals are reported as weight % of composition (see Data Set S1 in the supplemental material). The relative enrichment of select major and trace elements was determined for each sediment classification (Table 1). Groups with mean values above the cutoff limit were reported as enriched with an element.

**TABLE 1:** Sample means for sediment categories^*a*^

Element	Cutoff value	Sample mean for group:			
		1	2	3	4
Cl	450,000 mg/liter	**541,125**	70,342.86	**546,000**	383,000
Sr	200 ppm	106.375	**533**	122.8	**449.25**
Zn	10 ppm	**47.5**	**81.28571**	**40.2**	**11.5**
Mn	15 ppm	10.25	**37.85714**	9.2	10.25
Ba	20 ppm	1.75	**31.14286**	4.6	19
Ca	5%	1.9925	**8.867143**	2.376	**6.8675**
S	2%	1.5375	**6.435714**	1.824	**5.9275**
Mg	0.20%	0.12625	**0.422857**	0.086	0.1425
K	0.15%	**0.19625**	**0.204286**	0.112	0.1325
Al	0.10%	0.01375	**0.157143**	0.022	0.045
Fe	0.05%	0.02	**0.138571**	0.026	0.0425

a Boldface values indicate that an element is enriched in samples.

10.1128/mSphere.00378-19.10 DATA SET S1 Field information from sampling sites, geochemical data, and classification graphs (file name “SupplementalTable_Field, Mineralogical, ElementalData”). Download Data Set S1, XLSX file, 0.4 MB. Copyright © 2019 McGonigle et al. 2019 McGonigle et al. This content is distributed under the terms of the Creative Commons Attribution 4.0 International license.

Thin sections revealed group 1 (surface halite) consists of puffy fine efflorescent halite crystals, followed by larger halite crystals (up to 0.5 cm wide) with fluid inclusions. Group 1 samples are composed of predominantly halite with gypsum (2.5 to 27%) and trace amounts (<0.5%) of clay. Group 1 is relatively enriched in chloride, potassium, and zinc (from most to least abundant).

Group 2 (upper gypsum) consists of layered fine to medium sized gypsum grains. Group 2 samples contain gypsum (29 to 39%) and halite (6 to 22%). Group 2 samples contain the largest amount of clay and detrital minerals of any strata (0.3 to 1.7%). Reconstructed mineralogy only accounted for 41 to 61% of group 2’s mass. Group 2 is enriched in sulfur, calcium, magnesium, aluminum, potassium, iron, strontium, zinc, barium, and manganese.

Group 3 (lower halite) samples are cemented halite with porous vertical dissolution pipes. These pipes are typically 10 to 30 mm wide, but some are >1 cm. Elemental data from group 3 are similar to the data from group 1 (surface halite). Group 3 is enriched in chloride and zinc.

Group 4 (halite mixed with gypsum) consists of a chaotic halite supported framework (53 to 61%) made from vestigial dissolution pipes filled by pore space and gypsum (28 to 33%). Group 4 is enriched in calcium, sulfur, strontium, and zinc.

### Bacterial and archaeal community composition.

All sediment samples produced 1,845,510 merged paired reads with the bacterial primer set and 552,906 merged paired reads with the archaeal primer set. From these, DADA2 identified 2,740 amplicon sequence variants (ASVs) in the bacterial data set and 1,646 ASVs in the archaeal data set. DNA extraction yields and ASV counts per sample are shown in Table S1 in the supplemental material. These ASV counts were transformed with the DNA extraction yields with the procedure described in the methods to obtain biomass-weighted environmental abundances for downstream analyses. At most sites, the samples in groups 1 and 2 have higher DNA yields and ASV counts than the samples from group 3 or group 4. Samples from groups 1 and 2 were sequenced more successfully than those in other groups, particularly with archaeal primers.

10.1128/mSphere.00378-19.8 TABLE S1 DNA yields and ASV counts for each sample. Download Table S1, DOCX file, 0.02 MB. Copyright © 2019 McGonigle et al. 2019 McGonigle et al. This content is distributed under the terms of the Creative Commons Attribution 4.0 International license.

There were 1,552 ASVs (56% of the total ASVs) in the bacterial data set that were assigned archaeal taxonomy. The 515F/806R bacterial primers are known to amplify both bacteria and archaea, so we chose to include the archaeal ASVs in the bacterial data set rather than remove such a large proportion of the total sequence diversity (20–22). Most archaeal ASVs in the bacterial data set overlap classifications found in the archaeal data set, but the archaeal data set generally provided more specific classifications, particularly for the class *Thermoplasmata*.

All samples are dominated by *Halobacteria* from six different families (*Haloadaptaceae*, *Halobacteriaceae*, *Halococcaceae*, *Haloferacaceae*, *Halomicrobiaceae*, and J07HX5) and *Salinibacter* species (Fig. S3). Sequences most closely related to *Geitlerinema* sp. strain PCC 9228 and Deltaproteobacteria from the families *Bradymonadaceae* and *Desulfohalobiaceae* were also abundant in many samples. Less-dominant taxa generally increase in abundance with depth at most sites.

All samples in the archaeal data set are dominated by Halobacteria from 34 different classified genera (Fig. S4). *Haloferacaceae* and *Halomicrobiaceae* are the most abundant families. *Halapricum* and *Halomicroarcula* (both in *Halomicrobiaceae*) are the most dominant genera among the *Halobacteria*. ASVs classified to *Halobacteriales* from the family J07HX5 appear to be more abundant in the upper sediments. ASVs classified as *Halosulfurarchaeum* appear to be higher in relative abundance in upper gypsum samples. Sequences related to *Hadesarchaeaeota*, *Nanohaloarchaeia* (particularly from *Aenigmarchaeales*), *Bathyarchaeia*, *Thermoplasmata*, and *Methanomicrobia* appear to be more abundant in lower layers.

The ASVs in the bacterial data set were classified as belonging to *Halobacteria* representing 31 genera. As in the archaeal data set, *Haloferacaceae* and *Halomicrobiaceae* are the most abundant families and *Halapricum* and *Halomicroarcula* (both in *Halomicrobiaceae*) were the most dominant genera across all samples. In this data set, however, it appears that *Halomicroarcula* decreases in relative abundance with the depth of the sample. *Halorussus*, *Halorubellus*, and unclassified *Halobacteriales*, on the other hand, increase in relative abundance with the depth of the sample.

The most dominant ASVs related to cyanobacteria are classified as *Geitlerinema* sp. PCC 9228 and are more abundant in upper surface halite and upper gypsum layers. The relative abundances of *Acetothermia*, *Marinilabiaceae* (and other *Bacteroidales*), *Desulfovermiculus*, *Gemmatimonadetes*, *Phycisphaeraceae*, and *Thiohalorhabdus* are greater in lower layers.

Multivariate analysis of the beta diversity for the bacterial and archaeal data sets did not reveal any significant correlations between community compositions and location or sediment depth. (Fig. S5). Similarly, the alpha diversity, as measured by the Simpson index, did not exhibit any consistent patterns with location or sediment depth (Table S2). These results highlight the general lack of significant variation among the whole-community compositions prior to differential abundance analyses.

10.1128/mSphere.00378-19.9 TABLE S2 Simpson’s diversity index values for each sample in each dataset. Download Table S2, DOCX file, 0.01 MB. Copyright © 2019 McGonigle et al. 2019 McGonigle et al. This content is distributed under the terms of the Creative Commons Attribution 4.0 International license.

### Differential abundance.

Despite the lack of variation at a whole community level, we investigated whether specific ASVs are significantly enriched in one or more sediment classifications. Differential abundances of ASVs were calculated with edgeR (23) by comparing each of the sediment groups to each other (Fig. 1). For both data sets, comparisons were made between groups 1 and 2, between groups 2 and 3, and between groups 3 and 4. All comparisons were made with at least two samples per group.

**FIG 2:**
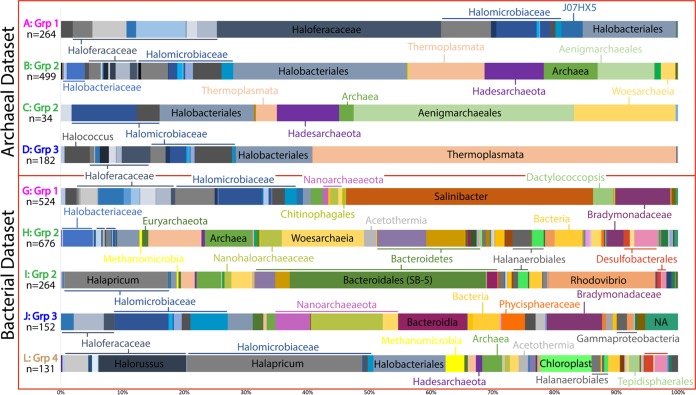
Abundances of ASVs enriched in each group, as determined by the differential abundance (edgeR) comparisons shown in Fig. 1. The numbers of ASVs making up the total plot are indicated below the comparison ID. A full legend can be found in Fig. S6.

In the bacterial data set, comparisons between groups 1 and 2 identified 524 ASVs, with significantly greater abundance (determined by a false discovery rate [FDR] of <0.05) in group 1 (surface halite), and 676 ASVs, with significantly greater abundance in group 2 (upper gypsum) (Fig. 2; see also Fig. S6). Diverse *Halobacteria* are the most abundant of the surface halite-enriched ASVs (41%). *Salinibacter* species are also highly abundant among the surface halite-enriched ASVs (40%). The remaining ASVs enriched in group 1 are related to the cyanobacteria *Dactylococcopsis* sp. strain PCC-8305, *Bradymonadaceae*, *Nanohaloarchaeota*, and unclassified members of *Chitinophagales* and *Archaea*. The enriched taxa become more diverse as the sediment transitions from surface halite to the upper gypsum layer. Taxa with a greater abundance in these group 2 sediments include *Thermoplasmata*, *Nanohaloarchaeaceae*, *Woesearchaeia*, *Acetothermia*, *Halanaerobium*, *Desulfovermiculus*, *Thiohalorhabdus*, *Parcubacteria*, *Planctomycetes*, *Gemmatimonadetes*, *Marinilabiaceae*, and other *Bacteroidetes*. *Methanomicrobia* are also more enriched in upper gypsum sediments, as well as unclassified bacterial and archaeal ASVs.

In the archaeal data set, comparisons between groups 1 and 2 identified 264 ASVs, with significantly higher abundance in group 1 (surface halite), and 499 ASVs, with significantly higher abundance in group 2 (upper gypsum). Diverse *Halobacteria* make up 99% of the abundance of the surface halite-enriched ASVs in samples from this group, with the majority of these related to *Haloferaceae*. The remaining enriched ASVs in group 1 belong to members of deep-sea euryarchaeotal groups (DSEG) and *Thaumarchaeota*. The ASVs enriched in group 2 are dominated by diverse *Halomicrobiaceae* and unclassified *Halobacteriales*. Other highly abundant upper gypsum-enriched ASVs grouped most closely with *Thermoplasmata*, *Hadesarchaeota*, *Aenigmarchaeales*, and unclassified *Archaea*.

The numbers of enriched ASVs identified by differential abundance comparisons between upper gypsum and deeper sediment layers are much lower. In the bacterial data set, comparing group 2 (upper gypsum) and 3 (lower halite) revealed 264 enriched ASVs in group 2, mostly from *Halapricum*, *Bacteroidetes* (specifically from the SB-5 group), and *Rhodovibrio*. Less-abundant upper gypsum-enriched ASVs include *Desulfobacterales*, *Methanomicrobia*, *Nanohaloarchaeaceae*, *Wosearchaeia*, *Halanerobiales*, and unclassified *Bacteria* and *Archaea*. The comparison found 152 group 3-enriched ASVs. The enriched *Halobacteria* in these sediments are heavily represented by *Halomicrobiaceae*. The other abundant lower halite-enriched ASVs are most closely related to *Nanoarchaeaeota*, *Bacteroidia*, *Phycisphaeraceae*, and *Bradymonadaceae*. *Coxiella* and other unclassified *Gammaproteobacteria* are also more enriched in these group 3 sediments.

In the archaeal data set, comparisons between groups 2 (upper gypsum) and 3 (lower halite) found only 34 ASVs that were more abundant in group 2 samples. The most abundant enriched ASVs were classified as *Aenigmarchaeales* and *Woesearchaeia*. Other upper gypsum-enriched ASVs were closely related to *Halomicrobiaceae* (particularly *Halomicroarcula*), *Halobacteriales*, *Thermoplasmata*, and *Hadesarchaeota*. There were 182 ASVs enriched in group 3 (lower halite) samples. The most abundant of these ASVs were classified as *Thermoplasmata*. The remaining ASVs are *Halobacteria* from *Halococcus*, *Haloferacaceae*, *Halomicrobiaceae*, and *Halobacteriales*.

In the archaeal data set, comparisons between group 3 (lower halite) and group 4 (lower halite and gypsum) samples found no enriched ASVs in either category. In the bacterial data set, these comparisons found 131 enriched ASVs in group 4 and none in group 3. The most abundant of these ASVs are most closely related to *Halapricum*. Other significantly enriched *Halobacteria* in these lower halite and gypsum layers include *Halorussus*, *Halobellus*, and unclassified *Halobacteriales*. ASVs related to *Methanomicrobia*, *Acetothermia*, *Chloroplast*, *Halanerobiales*, *Tepidisphaerales*, and *Desulfobacterales* are also enriched in these sediments.

## DISCUSSION

### The Bonneville Salt Flats hosts a robust microbial ecosystem.

Despite its barren appearance on the surface, the Bonneville Salt Flats (BSF) harbors a robust microbial ecosystem potentially capable of a range of metabolic capabilities, including phototrophy, sulfur and nitrogen cycling metabolisms, and heterotrophy. Although hypersaline systems can be harsh environments for life, they have been previously reported to contain high biomass (17, 24, 25). Our results indicate that BSF is no exception. If we assume 2 × 10^−6^ ng of DNA per cell, we estimate 10^7^ to 10^9^ cells per g of sample from our quantifications of environmental DNA. This is likely to be an overestimate because it is known that salt can preserve DNA, and our environmental DNA could include a significant extracellular fraction (26, 27). In addition, the DNA could also represent eukaryotic species not captured by our 16S rRNA primers. Nevertheless, the low range of our biomass estimate is consistent with other reported values around 10^6^ to 10^7^ cells ml^−1^ (17, 24, 25).

The surface halite and upper gypsum sediment layers (groups 1 and 2) have higher DNA yields than deeper samples (Table S1). These upper sediments have access to sunlight and oxygen while still retaining protection from UV radiation within the evaporite crusts. In addition, sulfur minerals in the upper gypsum layer likely provide an energy source for chemotrophic microbes living in these upper gypsum layers. Therefore, the upper gypsum layer is likely to have more abundant and more active phototrophic and chemotrophic microbial communities compared to deeper layers.

### A microbial community shift occurs along a mineralogical shift in the sediment.

Although the depth of each sediment layer varied per site, microbial communities seemed homogenous among samples within each sediment category. For example, at site 12B, the surface halite was only 3 mm, while at site 56 the surface halite was 25 mm (see Fig. S7 and Data Set S1 in the supplemental material). In spite of these differences in depth, these surface halite samples hosted remarkably similar archaeal and bacterial communities (Fig. S3 and S4). This trend is still evident even between the two samples with the greatest spread between sampling depths: site 12B, where the upper gypsum layer was sampled at a depth of 3 to 7 mm, and site 41, where the gypsum layer was sampled at a depth of 25 to 90 mm. Both of these samples appear similar in mineral classification and relative abundance of microbial taxa. These results suggest microbial communities shift along mineralogical rather than depth gradients at BSF.

Upper halite crusts (group 1) are dominated by heterotrophs from *Salinibacter* and *Halobacteria*. *Halobacteria* gain most of their energy through heterotrophy but are also capable of harnessing sunlight for supplemental ATP production with the specialized pigment bacteriorhodopsin (28–30). *Haloferacaceae* is the dominant family in these upper sediments. Key primary producers in the top halite layer are likely cyanobacteria, particularly *Dactylococcopsis* sp. strain PCC-8305, whose sequences are specifically enriched in these top layers, and the salt-tolerant alga *Dunaliella salina*. Although we did not perform 18S rRNA sequencing to characterize the eukaryotic community, one ASV had 100% identity to the 16S rRNA gene of the *Dunaliella* chloroplast. This ASV was absent at three sites: 12B, 67B, and 41. Sites 12B and 67B had the highest proportion of gypsum for samples in group 1. These sites are also located in a region with the thinnest surface halite (Fig. S7).

Thin sections show that surface halite (group 1) contains large pore spaces and fluid inclusions within the halite crystals (Fig. 1). This upper phototrophic community at BSF likely resides in tandem with heterotrophs in the pore spaces of the halite crystals or as a filamentous biofilm attached to the surfaces of halite crystals (31). Microbes in this surface layer may also be encapsulated within fluid inclusions. Members of this community are likely most active during the flooding stage of BSF, when upper halite dissolves and brine exists on the surface. They may become encapsulated as the brine precipitates into halite crystals during the evaporation period.

Our results indicate that more taxonomic groups are enriched in the upper gypsum sediments (group 2) found beneath the surface halite layer than in other types of sediment (Fig. 2). The higher surface area of clay minerals present in these samples likely provides a more suitable substrate for growth than the surface halite. *Bacteroidetes*, which are often associated with the rhizosphere in other less-extreme soils, are interestingly enriched in this layer (32). Microbes may be concentrated in this layer because gypsum, halite, and other evaporite minerals provide some protection from harsh environmental conditions (such as UV radiation and extreme temperature fluctuations) while still transmitting enough sunlight to support photosynthesis (33). Phototrophs enriched in upper gypsum sediments include members of *Rhodovibrio*, the purple nonsulfur bacteria. In this layer, we also observe abundant sequences related to *Geitlerinema* sp. PCC 9228, which has been shown in cultures to switch between oxygenic photosynthesis and anoxygenic photosynthesis using sulfide as an electron donor (34).

In general, bacterial taxa associated with sulfur metabolisms (e.g., *Thiohalorhabdus*, *Desulfovermiculus*, *Desulfobacterales*, *and Halanaerobium*) are enriched in the upper gypsum (group 2) layer. These results are strongly suggestive that the abundant sulfur minerals found in this layer (Table 1 and Data Set S1) are metabolized and cycled by the resident microbes. Interestingly, methanogens and acetogens, which often compete with sulfate-reducing bacteria, are also enriched in the upper gypsum layer (35). Methanogens in salt flats often utilize methylated compounds, rather than carbon dioxide, allowing them to coexist with acetogens and sulfate-reducing bacteria (36).

The lower halite and mixed halite and gypsum sediments (groups 3 and 4) contain chemoheterotrophic (*Thermoplasmata* and *Phycisphaeraceae*) and fermentative taxa, perhaps dependent on primary production of the phototrophic community in upper layers. *Bradymonadaceae* (whose type genus was recently described as a facultative anaerobe) and *Tepidisphaerales* (whose type genus was recently described as a facultative aerobe) become enriched again in these lower sediments, suggesting that the transition to a more anaerobic environment occurs at these depths ranging from 15 to 120 mm (37, 38). Aerobic heterotrophs from the *Proteobacteria*, such as Acinetobacter and *Oligoflexales*, are also enriched here, although they are low in abundance. This suggests that oxygen may not be completely depleted everywhere at these depths. The dissolution pipes and larger grain sizes found in the lower halite and lower halite mixed with gypsum layers may enable more air and fluid circulation in these sediments (Fig. S2).

### The microbial community of BSF is similar to previously described hypersaline ecosystems.

Only one culture-independent study of microorganisms at the BSF has been conducted previously (39). This study focused on the adjacent basin Pilot Valley but also included one sampling location at the BSF. The genus *Halolamina* was the most dominant member of the *Halobacteria* in this BSF sample. *Halolamina* sequences were also identified in all of our samples, but the most abundant *Halobacteria* genera in our data set were *Halomicroarcula* and *Halapricum* (Fig. S3 and S4). Culture-dependent studies at BSF have only been successful at isolating *Haloarcula*, *Halorubrum*, *Halobacterium*, and *Salicola* species (40, 41). We also found ASVs with low abundance most closely related to each of these species in our data sets.

The microbial diversity at BSF is comparable to that of other dry saline environments. Archaeal communities are often dominated by members of *Halobacteria* (42–44). Bacterial communities are often dominated by *Bacteroidetes* (*Salinibacter*) and *Proteobacteria* (45–47). Members from *Crenarchaeota*, *Gemmatimonadetes*, *Verrucomicrobia*, SRB from Deltaproteobacteria, and *Clostridia* are also commonly reported (48–50). We found ASVs at BSF that are closely related to all of these commonly reported species (Fig. S3 and S4). *Euhalothece* is the most commonly reported dominant cyanobacterium in hypersaline environments (31, 45, 47, 49–52). All of our *Cyanobacteria* ASVs classified within the oxygenic phototrophic class *Oxyphotobacteria*, but we found the oxygenic/anoxygenic *Geitlerinema* sp. PCC 9228 to be the most dominant *Cyanobacteria* at BSF.

Interestingly, many of these same microbial taxa have also been reported in hypersaline lake environments, such the Great Salt Lake, Lake Chaka, and the Salton Sea (51–57), suggesting physiological and/or ecological connections between hypersaline aquatic and evaporite systems.

### Conclusions.

The Bonneville Salt Flats hosts thriving microbial communities dominated by diverse *Halobacteriaceae* and *Salinibacter* species that are adapted to the challenges of this extreme ecosystem. Many of the archaeal and bacterial taxa are most abundant beneath the halite crust within the upper gypsum sediments, where microbes are likely metabolizing sulfur-containing minerals and can still rely on light penetration for phototrophy.

This study represents the first comprehensive culture-independent characterization of the microbial communities inhabiting the Bonneville Salt Flats, and future work in this understudied ecosystem could address gaps in knowledge, such as which microbes in this ecosystem are crucial for the cycling of nutrients like sulfur and nitrogen, whether haloarchaea are dependent on light filtered through the surface halite crust, how the microbial community shifts with seasonal desiccation and flooding stages, and whether anthropogenic use of this environment for racing and brine mining changed the microbial composition of the ecosystem.

Answering these questions would extend our understanding of the presence and preservation of life not only in extreme environments but also in any environment where salt deposits are formed. Halite crystal growth bands can preserve microbial life present in the original air-water and water-sediment interfaces where the salt formed thousands to hundreds of millions of years ago (13, 58). Evaporite ecosystems may even reflect life on Earth prior to the Cambrian explosion (15). Furthermore, because evaporite deposits on other planetary bodies, such as Mars, are evidence of past or present water (59–62), further advances in the characterization of live and preserved microbes in salt deposits will have implications for the development of extraterrestrial life detection strategies.

## MATERIALS AND METHODS

### Sampling location and collection.

Samples were collected from eight pits dug with sterile tools in September 2016 **(**Data Set S1 and Fig. S1). Distinct sediment layers visually identified by color and textural changes were sampled at each site. Samples were immediately placed on ice and transported within 12 h to the University of Utah, where they were stored at –80°C until microbiological analyses were performed in the following 6 months.

10.1128/mSphere.00378-19.1 FIG S1 Sampling locations and field photos of sampling sites. Black lines indicate approximate location of the racetrack at the time of sampling. Download FIG S1, TIF file, 1.8 MB. Copyright © 2019 McGonigle et al. 2019 McGonigle et al. This content is distributed under the terms of the Creative Commons Attribution 4.0 International license.

10.1128/mSphere.00378-19.2 FIG S2 Thin sections used to create representative textural diagrams for Fig. 1. All thin sections are oriented with the stratigraphic up at the top. Note that a yellow box is used to highlight the area of focus for the image on right side of each box. All images on the left and right sides of each box share the scale bar as shown in panel A. (A) Complete thin section from site D-35. This sample comprises groups 1, 2, and 3 (left). Efflorescent and cumulate halite with thin gypsum layers are shown on the right. (B) Thin section from near site D-41 comprising groups 1 and 2 (left). Distinct layers of gypsum with organic-rich layers can be seen; the clear area running through the sample is a sampling artifact (right). Thin sections from panels C to E have a pink epoxy that was used to highlight pore space. (C) Thin section from site D-56 comprised by group 3 (left). Dissolution pipe with high porosity and permeability is shown on the right. (D) Thin section from site D-12B comprising group 4. Note the large amount of halite with remnant dissolution pipes that are mostly filled by gypsum but retain some porosity (left and right). Download FIG S2, TIF file, 2.7 MB. Copyright © 2019 McGonigle et al. 2019 McGonigle et al. This content is distributed under the terms of the Creative Commons Attribution 4.0 International license.

10.1128/mSphere.00378-19.3 FIG S3 Taxonomy of all ASVs from bacterial dataset in all samples. Download FIG S3, TIF file, 1.3 MB. Copyright © 2019 McGonigle et al. 2019 McGonigle et al. This content is distributed under the terms of the Creative Commons Attribution 4.0 International license.

10.1128/mSphere.00378-19.4 FIG S4 Taxonomy of all ASVs from archaeal dataset in all samples. Download FIG S4, TIF file, 0.6 MB. Copyright © 2019 McGonigle et al. 2019 McGonigle et al. This content is distributed under the terms of the Creative Commons Attribution 4.0 International license.

10.1128/mSphere.00378-19.5 FIG S5 (A) Multivariate analysis of bacterial dataset created in phyloseq using the Bray-Curtis beta diversity index. (B) Multivariate analysis of archaeal dataset created in phyloseq using the Bray-Curtis beta diversity index. Download FIG S5, TIF file, 0.7 MB. Copyright © 2019 McGonigle et al. 2019 McGonigle et al. This content is distributed under the terms of the Creative Commons Attribution 4.0 International license.

10.1128/mSphere.00378-19.6 FIG S6 ASVs with greater relative abundance in each group as determined by differential abundance (edgeR) comparisons noted in Fig. 1. Download FIG S6, TIF file, 1.2 MB. Copyright © 2019 McGonigle et al. 2019 McGonigle et al. This content is distributed under the terms of the Creative Commons Attribution 4.0 International license.

10.1128/mSphere.00378-19.7 FIG S7 Location of sampling sites and surface halite thickness across the Bonneville Salt Flats time of sampling (10). Note that this surface halite classification, while similar to group 1, is not equivalent, due to the coarser vertical resolution. Download FIG S7, TIF file, 1.3 MB. Copyright © 2019 McGonigle et al. 2019 McGonigle et al. This content is distributed under the terms of the Creative Commons Attribution 4.0 International license.

### Elemental and sedimentological analysis.

A portion of each homogenized sample was separated and analyzed for major and trace elements by ActLabs. Major and trace elements were analyzed via Aqua Regia ICP (Inductively Coupled Plasma, method 1E3). Anions were analyzed via ion chromatography (method 6B). Using these elemental data, along with visual characteristics, each sample was grouped into one of four sediment categories. Elemental concentrations were used to reconstruct relative weight mineralogy based upon stoichiometry of gypsum, halite, silicates, and other detrital minerals. Elemental concentrations beyond detection limits were derived using this method.

In addition to these samples, additional blocks of representative material were collected at the same time of sampling from sites 56, 35, and 12B. These blocks were processed by Wagner Petrographic to create petrographic thin sections for visual microscopy analysis. Blocks were desiccated to remove any pore waters and impregnated with clear or colored epoxy. Several thin sections were made from each block by cutting 50- by 75-mm blocks and mounting them on glass slides. Samples were cut to thicknesses ranging from 0.5 to 2 mm. The samples were then analyzed at the University of Utah using an Olympus BX53M microscope with ×1,000 magnification and a Zeiss M2 petroscopic microscope with an Axiocam camera and Zen 2 Pro software.

### DNA extraction and sequencing.

DNA was extracted from salt crust samples using a protocol from Brazelton et al. (63) modified for highly saline material. The modified protocol is available on our lab’s website (https://baas-becking.biology.utah.edu/data/category/18-protocols) and summarized here. Sediment samples were crushed and homogenized with a sterile mortar and pestle, and 0.25-g subsamples were placed in a DNA extraction buffer containing 0.1 M Tris, 0.1 M EDTA, 0.1 M KH_2_PO_4_, 0.8 M guanidium HCl, and 0.5% Triton X-100. For lysis, samples were subjected to one freeze-thaw cycle, incubation at 65°C for 30 min, and beating with 0.1- mm glass beads in a Mini-Beadbeater-16 (Biospec Products). Purification was performed via extraction with phenol-chloroform-isoamyl alcohol, precipitation in ethanol, washing in Amicon 30K Ultra centrifugal filters, and final cleanup with 2× SPRI beads (64). DNA quantification was performed with a Qubit fluorometer (Thermo Fisher).

Archaeal and bacterial 16S rRNA gene amplicon sequencing was conducted by the Michigan State University genomics core facility. The V4 region of the bacterial 16S rRNA gene was amplified with dual-indexed Illumina fusion primers with the 515F/806R primers, as described by Kozich et al. (65). The V4 region of the archaeal 16S rRNA gene was amplified with the A519F/Arch958R primers (66). Amplicon concentrations were normalized and pooled using an Invitrogen SequalPrep DNA Normalization Plate. After library QC and quantitation, the pool was loaded on an Illumina MiSeq v2 flow cell and sequenced using a standard 500 cycle reagent kit. Base calling was performed by Illumina real-time analysis (RTA) using software v1.18.54. The RTA output was demultiplexed and converted to fastq files using Illumina Bcl2fastq v1.8.4.

### Analysis of 16S rRNA amplicon data.

Quality screening and processing of all 16S rRNA amplicon sequences was conducted with the DADA2 R package (67). Using this package, primer contaminants and chimeras were removed, reads were trimmed and filtered based on quality, and sequence variants likely to be derived by sequencing error were identified. The final amplicon sequence variants (ASVs) are considered to be true variants and are analyzed in the same method as traditional operational taxonomic units. The taxonomic classification of all ASVs was performed with the DADA2 package and the SILVA reference database (NRv132).

Sequence counts for each ASV were transformed with a biomass correction by multiplying the proportion of each ASV’s contribution to each sample’s total sequence count by the estimated number of total cells in that sample. The total cells were estimated by using the total amount (in ng) of extracted DNA (as measured by the Qubit fluorometer) and an assumption of 2 × 10^−6^ ng of DNA per cell. All downstream analyses were performed with these biomass-weighted relative abundances. Differential abundance of ASVs in each layer was performed using edgeR (23) and phyloseq (68). edgeR was used to determine the FDR using the Benjamini-Hochberg method. An ASV was considered to be significantly enriched in a layer if its differential abundance passed an FDR threshold of 0.05. Multivariate analysis was done using phyloseq (68), and Bray-Curtis and Simpson indices were calculated using vegan (69).

### Data availability.

All sequence data are publicly available at the NCBI Sequence Read Archive under BioProject PRJNA522308. All SRA metadata, full supplementary material, and protocols are archived at https://doi.org/10.5281/zenodo.2827066. All custom software and scripts are available at https://github.com/Brazelton-Lab.
