# Supplementary material for: Robust Archaeal and Bacterial Communities Inhabit Shallow Subsurface Sediments of the Bonneville Salt Flats
Source: mSphere. 2019 Aug 28;4(4):e00378-19. doi: 10.1128/mSphere.00378-19 (PMC6714890; doi:10.1128/mSphere.00378-19)
Supplement: TABLE S1 [file mSphere.00378-19-st001.docx]

| **Site ID** | **DNA Yield (ng/g sample)** | | | | **Total ASVs (bacterial primers)** | | | | **Total ASVs (archaeal primers)** | | | |
| --- | --- | --- | --- | --- | --- | --- | --- | --- | --- | --- | --- | --- |
|  | **G1** | **G2** | **G3** | **G4** | **G1** | **G2** | **G3** | **G4** | **G1** | **G2** | **G3** | **G4** |
| **12B** | 12840 | 2528 | - | 50.4 | 65053 | 67854 | - | - | - | - | - | 73436 |
|  |  | 2192 |  |  |  | 106198 |  |  |  | - |  |  |
| **67B** | 5120 | 182.4 | - | 100 | 85544 | 84097 | - | - | - | - | - | - |
| **29** | 801.6 | 2312 | 0.436 | - | 89366 | 105104 | 30302 | - | 40238 | 44315 | - | - |
| **33** | 94.4 | 16000 |  | 224 | 107635 | 68446 | - | 66105 | 56410 | 54253 | - | 49224 |
| **35** | 449.6 | - | 7852 | - | 96715 | - | 74831 | - | 54148 | - | - | - |
|  |  |  | 2256 |  |  |  | 109756 |  |  |  | 49935 |  |
| **56** | 816 | - | 4720 | 252.8 | 92117 | - | 100904 | 68563 | - | - | 39666 | - |
|  |  |  | 949.6 |  |  |  | 95481 |  |  |  | - |  |
| **41** | 1026.4 | 1258 | - | - | 84040 | 81330 | - | - | 41365 | - | - | - |
| **46** | 2696 | 12600 | - | - | 85504 | 80565 | - | - | - | 49916 | - | - |
| (-) indicates a sample which failed sequencing or wasn’t sampled | | | | | | | | | | | | |
