# Supplementary material for: Robust Archaeal and Bacterial Communities Inhabit Shallow Subsurface Sediments of the Bonneville Salt Flats
Source: mSphere. 2019 Aug 28;4(4):e00378-19. doi: 10.1128/mSphere.00378-19 (PMC6714890; doi:10.1128/mSphere.00378-19)
Supplement: TABLE S2 [file mSphere.00378-19-st002.docx]

|  | Bacterial Dataset | Archaeal Dataset |
| --- | --- | --- |
| 12B-1 | 0.9637356 | - |
| 12B-2 | 0.9370023 | - |
| 12B-3 | 0.9768435 | - |
| 12B-4 | - | 0.9505377 |
| 67B-1 | 0.9691345 | - |
| 67B-2 | 0.9824577 | - |
| 29-1 | 0.9822437 | 0.9878397 |
| 29-2 | 0.9799543 | 0.9883126 |
| 29-3 | 0.9873348 | - |
| 33-1 | 0.9693695 | 0.9701907 |
| 33-2 | 0.9843773 | 0.9877309 |
| 33-3 | 0.9883092 | 0.9840068 |
| 35-1 | 0.9729288 | 0.9734849 |
| 35-2 | 0.9876492 | - |
| 35-3 | 0.9876442 | 0.9875751 |
| 56-1 | 0.9852115 | - |
| 56-2 | 0.9805659 | 0.9878431 |
| 56-3 | 0.9877158 | - |
| 56-4 | 0.9872632 | - |
| 41-1 | 0.9831951 | 0.9886681 |
| 41-2 | 0.9811539 | - |
| 46-1 | 0.9550911 | - |
| 46-2 | 0.9829102 | 0.9926546 |
