# Supplementary figures and images for: Robust Archaeal and Bacterial Communities Inhabit Shallow Subsurface Sediments of the Bonneville Salt Flats
Source: mSphere. 2019 Aug 28;4(4):e00378-19. doi: 10.1128/mSphere.00378-19 (PMC6714890; doi:10.1128/mSphere.00378-19)

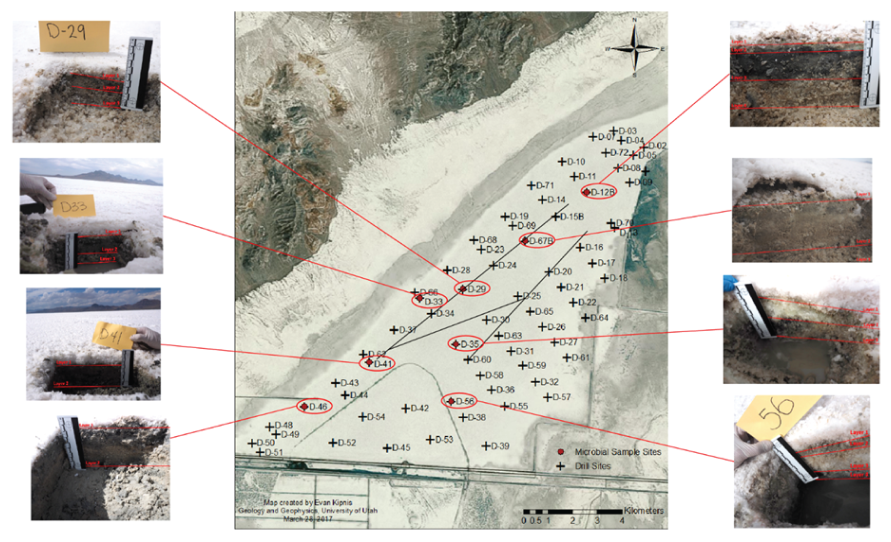

Supplement: FIG S1 [file mSphere.00378-19-sf001.tif]

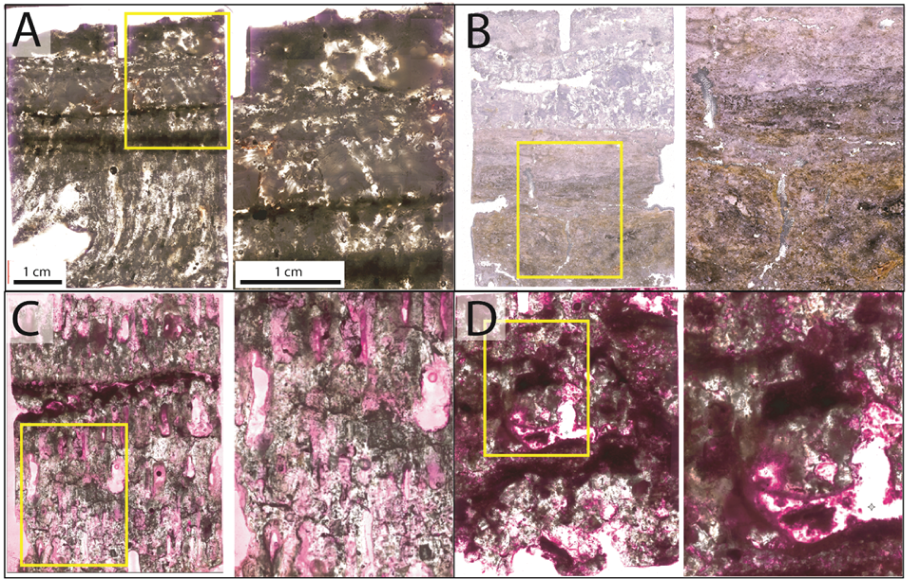

Supplement: FIG S2 [file mSphere.00378-19-sf002.tif]

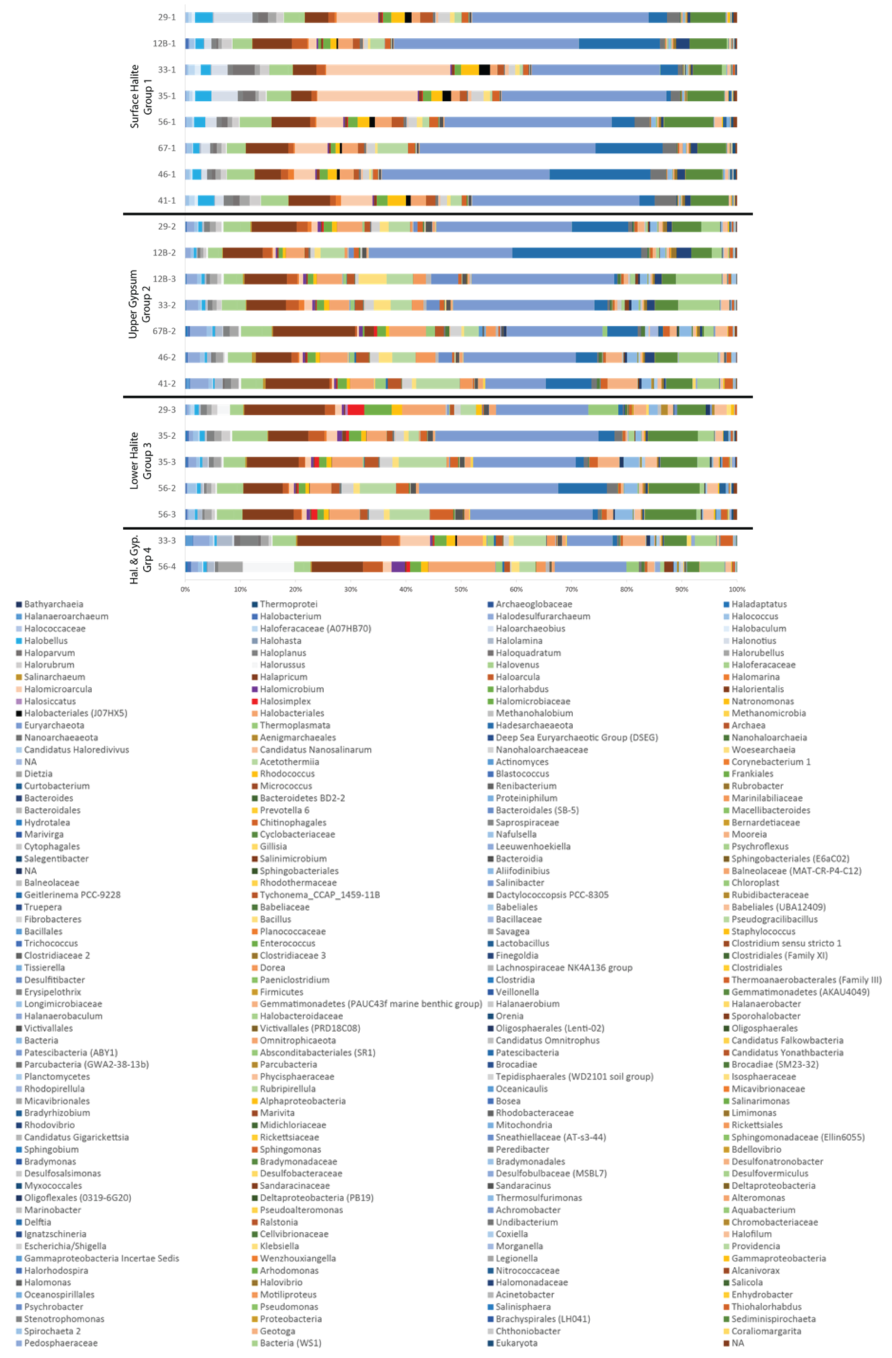

Supplement: FIG S3 [file mSphere.00378-19-sf003.tif]

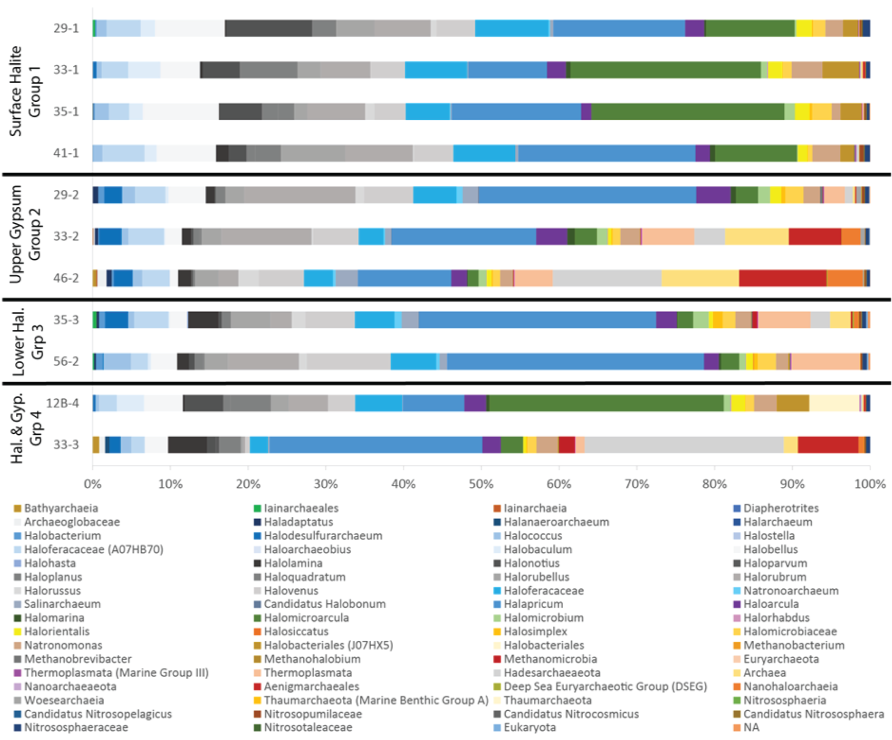

Supplement: FIG S4 [file mSphere.00378-19-sf004.tif]

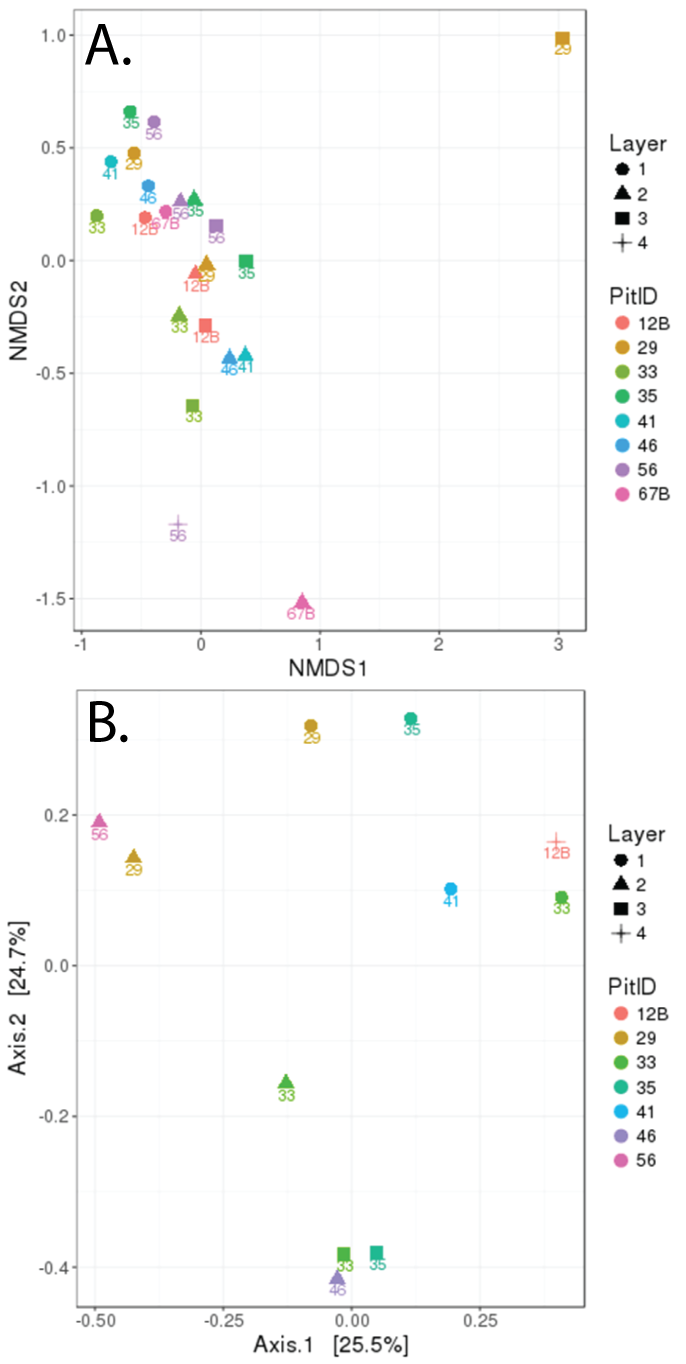

Supplement: FIG S5 [file mSphere.00378-19-sf005.tif]

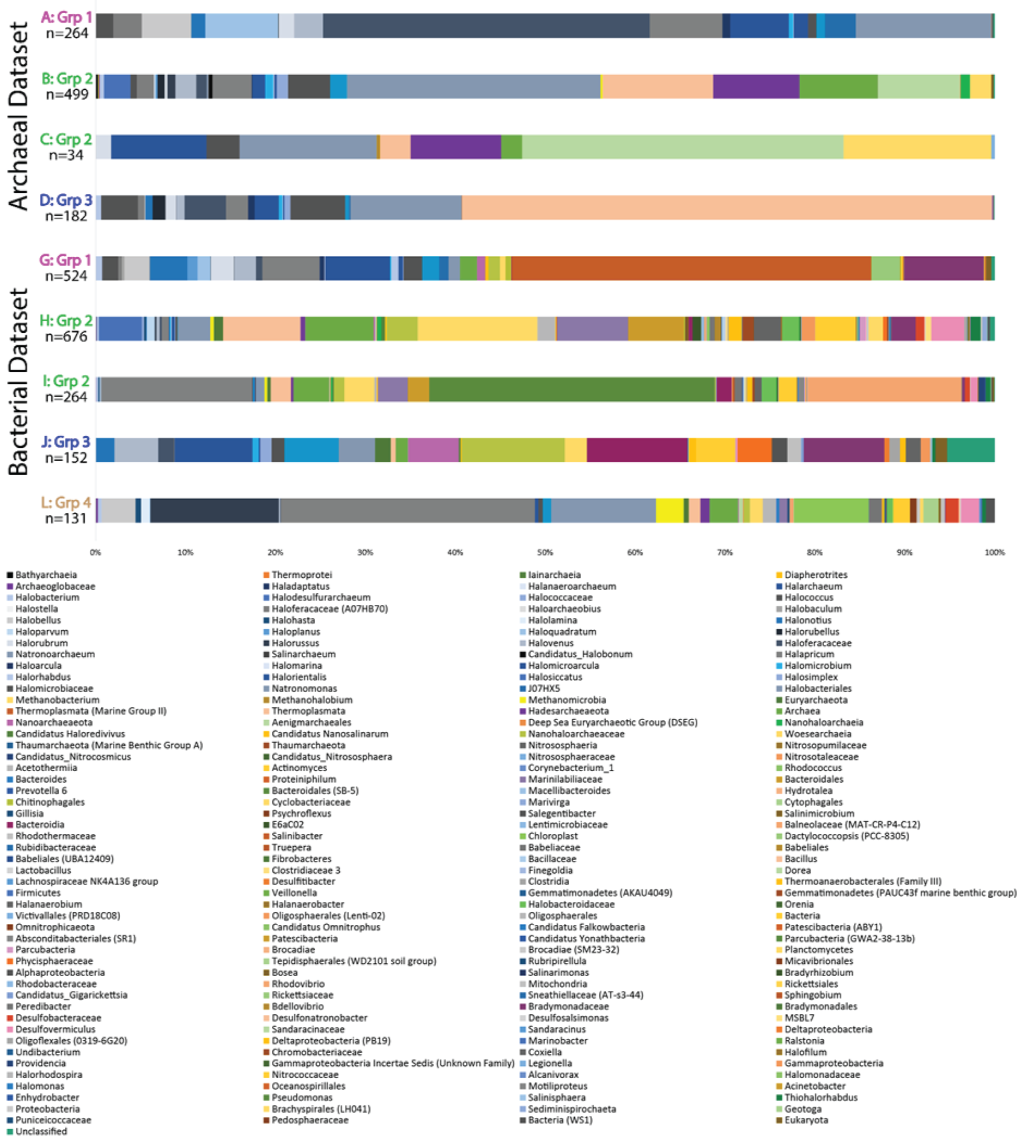

Supplement: FIG S6 [file mSphere.00378-19-sf006.tif]

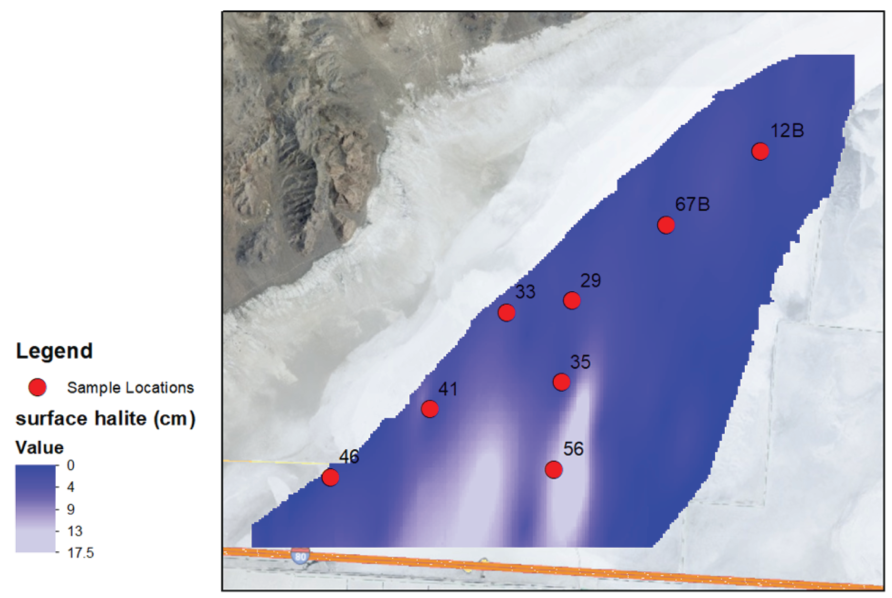

Supplement: FIG S7 [file mSphere.00378-19-sf007.tif]
